# Application of X-ray Microcomputed Tomography for the Static and Dynamic Characterization of the Microstructure of Oleofoams

**DOI:** 10.1021/acs.langmuir.1c03318

**Published:** 2022-01-20

**Authors:** Lorenzo Metilli, Malte Storm, Shashidhara Marathe, Aris Lazidis, Stephanie Marty-Terrade, Elena Simone

**Affiliations:** † School of Food Science and Nutrition, Food Colloids and Bioprocessing group, University of Leeds, Woodhouse Lane, Leeds LS29JT, U.K.; ‡ Diamond Light Source Ltd., Harwell Science and Innovation Campus, Didcot OX110DE, U.K.; § Helmholtz-Zentrum hereon, Max-Planck-Str 1, 21502 Geesthacht, Germany; ∥ Nestlé Product Technology Centre Confectionery, Haxby Road, York YO31 8TA, U.K.; ⊥ Nestlé Research, Vers-chez-les-Blanc, Lausanne 26 1000, Switzerland; # Department of Applied Science and Technology (DISAT), Politecnico di Torino, Corso Duca degli Abruzzi 24, Torino 10129, Italy

## Abstract

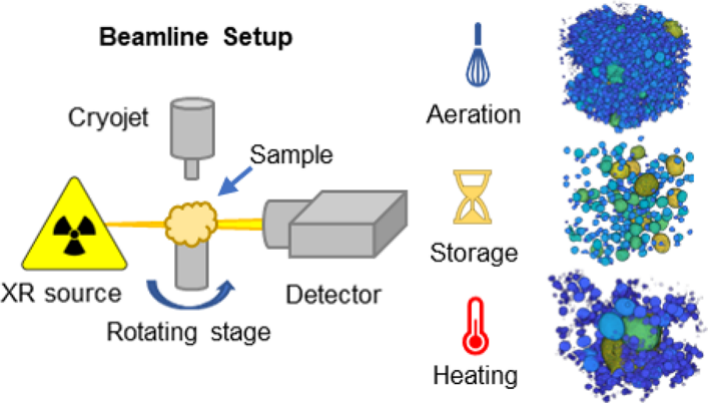

Oleofoams are a novel, versatile, and biocompatible soft material that finds application in drug, cosmetic or nutraceuticals delivery. However, due to their temperature-sensitive and opaque nature, the characterization of oleofoams’ microstructure is challenging. Here, synchrotron X-ray microcomputed tomography and radiography are applied to study the microstructure of a triglyceride-based oleofoam. These techniques enable non-destructive, quantitative, 3D measurements of native samples to determine the thermodynamic and kinetic behavior of oleofoams at different stages of their life cycle. During processing, a constant bubble size distribution is reached after few minutes of shearing, while the number of bubbles incorporated keeps increasing until saturation of the continuous phase. Low amounts of solid triglycerides in oleofoams allow faster aeration and a more homogeneous microstructure but lower thermodynamic stability, with bubble disproportionation and shape relaxation over time. Radiography shows that heating causes Ostwald ripening and coalescence of bubbles, with an increase of their diameter and sphericity.

## Introduction

Oleofoams are an emerging type of soft matter with remarkable potential for application in pharmaceuticals, cosmetic, care, and food products.^1,2^ They comprise an oil continuous phase and a dispersed gas phase that is stabilized by adsorbed solid particles or molecular surfactants. In contrast to aqueous foams, their production is more challenging, due to the limited availability of suitable stabilizers for air/oil interfaces.^3,4^ Nevertheless, over the last 5 years, some publications appeared, which describe the use of fat crystals to produce oleofoams with a high air volume fraction and outstanding thermodynamic stability, up to several months.^1,2^

Highly stable oleofoams are promising materials for pharmaceuticals and cosmetic formulations as the oil continuous phase can dissolve and deliver lipophilic drugs. Moreover, the presence of gas bubbles enhances the permeation within the skin layer and improves customer acceptability compared to other solvents.^5,6^ Oleofoams can also be emulsified to produce air-in-oil-in-water systems (A/O/W), where hydrophilic and lipophilic active ingredients can be simultaneously delivered.^7^ A further advantage in the use of oleofoams is the absence of synthetic surfactants, which are perceived negatively by consumers and associated with negative health effects.^8^ The use of oleofoams in food is particularly compelling, as they can be used to reduce the calorific density of fat-based foods and provide novel, attractive mouthfeel to consumers due to their aerated nature.^9^ The industrial interest in oleofoams resulted already in several patents for their use as fat-replacers in baked goods and to enhance drug delivery in water-free formulations.^10−12^ Finally, understanding the foaming of crude oil and its stability is a relevant topic in the petroleum industry.^13,14^ Despite their wide potential, research on the properties of oleofoams is still scarce compared to their aqueous counterparts.

Solid-stabilized oleofoams are typically fabricated from the aeration of a dispersion of fat crystals in a liquid oil phase, also termed oleogel.^15−25^ Fat crystals stabilize these foams through a Pickering mechanism and by forming a three-dimensional network in the continuous phase.^9^ Most of the research on oleofoams has focused on understanding the relationship between the properties of crystals within the oleogel (size, shape, polymorph) and the resulting foamability and foam stability in the whipped state.^1,17,20,24^

What is still missing is a clear understanding of the complex relationship between formulation, processing conditions, material microstructure, and macroscopic properties of oleofoams, including their thermodynamic stability.^2,27,28^ The reason for this gap in knowledge is that the 3D characterization of oleofoams in their native state is extremely challenging. In fact, these materials are optically opaque, deform under small level of shear, and display a range of melting points close to room or body temperature.

Nevertheless, understanding the process–structure–function relationship of oleofoams is essential for fine-tuning the properties of the desired aerated material, in improving the design of the unit operations required to manufacture oleofoams, in better estimating the shelf-life of consumer products, and finally in controlling the responsiveness of oleofoams to stimuli, such as changes in temperature or application of shear.^26^

The thermodynamic stability of the microstructure of oleofoams is normally investigated by microscopy or by indirect measurements (e.g., oil drainage, texture analysis).^16,17,19,21,29^ Microscopy involves some degree of sample preparation and provide only 2D information, leading to artifacts in the observed microstructure, and a limited understanding of some three-dimensional features.^23,30−32^

In this work, synchrotron-radiation X-ray microcomputed tomography (XCT) and X-ray radiography (XRR) were applied to study the three-dimensional microstructure of oleofoams in their native state. These techniques allowed quantitative estimation of the number, size, and shape distributions of air bubbles in oleofoams, together with the thickness of the continuous phase, which cannot be measured accurately with any bidimensional microscopy techniques. Such parameters were then used to study the evolution of the microstructure of oleofoams during processing (aeration), storage, and destabilization upon temperature cycling, which mimics topical administration.

As a model system, cocoa butter-based oleofoams were evaluated. Cocoa butter can be used as a source of crystalline fat for oleogelation of vegetable oils, as recently demonstrated by Metilli et al.^17^ As the foam properties (foamability, rheology, and resistance to drainage) were mainly affected by the amount of solid fat in the oleogel precursor, in this study, samples with either relatively low (15% w/w) or high (30% w/w) cocoa butter content were investigated and compared. To the best of the knowledge of the authors, this publication is the first providing information on the three-dimensional, native microstructure of oil-based foams in both static and dynamic conditions.

## Experimental Section

### Sample Preparation

Mixtures of cocoa butter (CB) and high oleic sunflower oil (HOSO) were prepared by melting CB at 65 °C and adding it to HOSO at the same temperature in a concentration of either 15% or 30% w/w. The mixtures were then cooled to obtain a dispersion of fat crystals in oil (oleogel) and then aerated to produce an oleofoam. The crystallization was carried out in a 2 L jacketed metal vessel, which was connected to a Huber Ministat 250 thermostat (Huber, Germany) for temperature control. The sample temperature was monitored with a Pt-100 temperature probe immersed in the vessel. The sample was maintained under shear (200 rpm) using a DLH overhead stirrer (VELP Scientifica, Italy) equipped with an anchor-shaped mixer (8 cm diameter). The samples, termed “15S” for the 15% w/w CB in HOSO and “30F” for the 30% w/w CB in the HOSO mixture, were cooled from 65 to 0 °C at a nominal cooling rate of −0.10 and −0.75 °C/min, respectively. The samples were subsequently aerated using a planetary mixer (model 5KPM50, Kitchenaid, USA) with a constant shear rate (250 rpm). The oleogels were whipped for a total time of 30 min, collecting samples every 5 min to study the effect of aeration time.^17^ At each step, the overrun (i.e., the increase in the sample volume) was calculated with the technique most commonly known as the cup method, where the sample is weighed in a cup of known volume. The overrun is then determined through eq 1
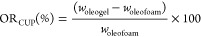
1where *w*_oleogel_ and *w*_oleofoam_ are the weight of the un-whipped oleogel and the weight of the oleofoam, respectively. The samples were imaged with XCT shortly after being whipped, or after 3 and 15 months of storage at 20 °C.

### Beamline Setup

The samples were analyzed at the I13-2 beamline at Diamond Light Source synchrotron (Didcot, UK), using a pink beam source with a mean energy of 27 keV (σ_*E*_ = 5 keV). The 2D projections for tomography and radiography were acquired with a PCO edge 5.5 CMOS camera (2560 × 2160 pixels). The camera objective used was a4×, with effective optical magnification of 8× and a pixel size of 0.8125 μm. A small amount of samples (approximately 1 mm^3^) was placed on top of a toothpick glued to the base of a cryocap. The samples were immersed in liquid nitrogen (−196 °C) and installed on the tomography rotating stage. The sample temperature was controlled with a Cryojet device (Cryojet XL, Oxford Instruments, UK) and set to −40 °C during the tomography acquisitions. The exposure time for each X-ray projection was set to 100 ms for 1001 projections, for a total acquisition time of 5 min.^37^ Each acquisition was carried out in triplicates on every sample.

### Time–Resolved XRR

Samples were subjected to heating, and the changes in their microstructure were studied using both X-ray tomography and radiography. The thermal treatment involved heating the sample from 20 °C to the melting temperature (*T_m_*) of the sample (25 °C for 15S and 27 °C for 30F), and holding at *T_m_* for 2 min. Afterward, the sample was cooled to 0 °C at −6 °C/min and maintained at such temperature for 5 min. Radiography images were collected every 0.5 s during heating and cooling, and their intensity was normalized according to eq 2:
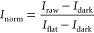
2where *I*_norm_ is the normalized pixel intensity of the analyzed image, *I*_raw_ is the pixel intensity of the sample image (projection), and *I*_dark_ and *I*_flat_ are the averaged pixel intensities of 20 dark field (instrument background) and 20 flat field (beam intensity distribution) images, respectively. The normalized radiography images were converted to a difference image stack, where each image is obtained from the absolute difference of pixel intensity between the i*-th* and the i + 1*-th* frame. The difference image stack was then analyzed with principal component analysis (PCA), using the *pca* function in MATLAB R2021a (Mathworks, USA). The score of the first principal component was plotted versus the sample temperature to detect the onset of microstructural destabilization observed by XRR. Radiography data were compared with differential scanning calorimetry (DSC) measurements obtained with a TA 8000 calorimeter (TA Instruments, USA). This onset temperatures obtained were compared with the TA Universal Analysis software (TA Instruments, USA). The 15S and 30F samples were heated up from 10 to 65 °C at a rate of 5 °C/min. The onset of the main melting endotherm peak was compared with the onset temperature of destabilization from XRR.

### Tomographic Reconstruction and Image Post-Processing

The tomographic dataset was processed using the *savu* framework developed at the Diamond Light Source.^40^ The projections were corrected for dark image and flat-fields (see above) and a ring-removal algorithm was applied.^41^ A Paganin-filter was applied to enhance the contrast. The tomography reconstruction was performed using the *Gridrec* reconstruction algorithm from the TomoPy software package, a python-based, open-source framework.^42,43^ The 3D volumes were then processed using ImageJ (National Institute of Health, USA). At least five volumes of interest (VOIs) of 500 × 500 × 500 μm^3^ were selected in each sample. The VOIs were then filtered using a 3D median filter, converted to binary images with Otsu thresholding, and segmented using a 3D Euclidean distance map.

Bubbles were counted using the BoneJ plugin, and their volume (V) and surface area (A) were measured.^44^ The air volume fraction was also measured (φ_air_), and used to calculate the sample overrun (OR_XCT_). Finally, BoneJ was used also to calculate the oleogel thickness, which is expressed as the volume of the sphere of maximum diameter that can be fitted in the oleogel phase.^45^ The descriptors of the sample microstructure used in this work are summarized in Table A, which can be found in the Appendix. A bubble cut-off diameter was set to 2.5 μm, to avoid noise caused by the voxel resolution limit (0.8125 μm). The number of bubbles counted for each sample was between 20,000 and 60,000, depending on its conditions (fresh, stored, or heated).

## Results and Discussion

### Evolution of Oleofoams’ Microstructure during Aeration

**Figure 1:**
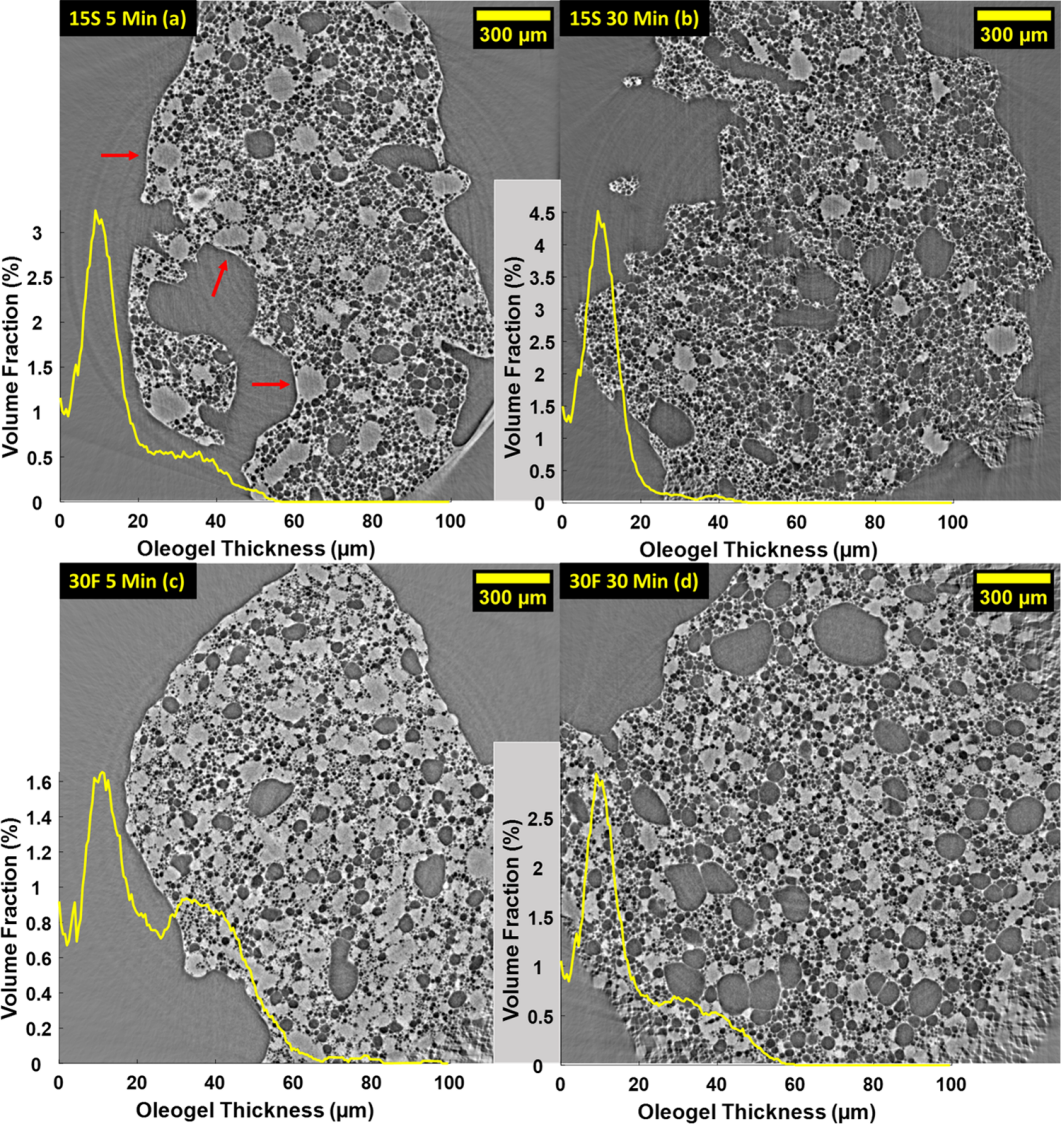
Tomographic slice of a 15S sample after 5 and 30 min of aeration (a, b) compared with a 30F sample after 5 and 30 min of aeration (c, d). The distribution of the oleogel thickness (yellow) is overlaid on the respective samples’ images. Large oleogel fragments are highlighted with a red arrow. Artifacts in the corner are due to the limited information in these regions; hence, they are not included in the analysis.

Figure 1 shows the microstructure of samples 15S and 30F at two different stages of the aeration process.

Slices of the reconstructed volumes, represented in a 2D plane, showed a clear distinction between the gas phase (dark gray pixels) and the continuous phase (light gray pixels) for all samples. The continuous phase in the oleofoam is the CB-HOSO oleogel,^17^ which comprises both the fat crystal network (CB) and the entrapped oil (HOSO). The thickness of the oleogel, calculated from the tomography data, is shown as a volume distribution for the samples. This parameter is fundamental in the study of the oleofoam microstructure and in the study of its relationship to their rheology and stability. It contributes, together with the air phase, to the viscoelastic profile of the oleofoams; furthermore, the continuous lipid phase has a significant role in stabilizing the air bubbles against coalescence, as demonstrated by several authors.^9,15,17,29^ Furthermore, the fat crystal network present in the oleogel phase also affects the oil binding capacity of oleofoams, preventing liquid drainage from the structure.^33^ The evolution of the continuous phase thickness during storage is also of great importance, as lipid crystals dispersed in the oleogels are subjected to Ostwald ripening and sintering (formation of crystal bridges).^17,31^

**Figure 2:**
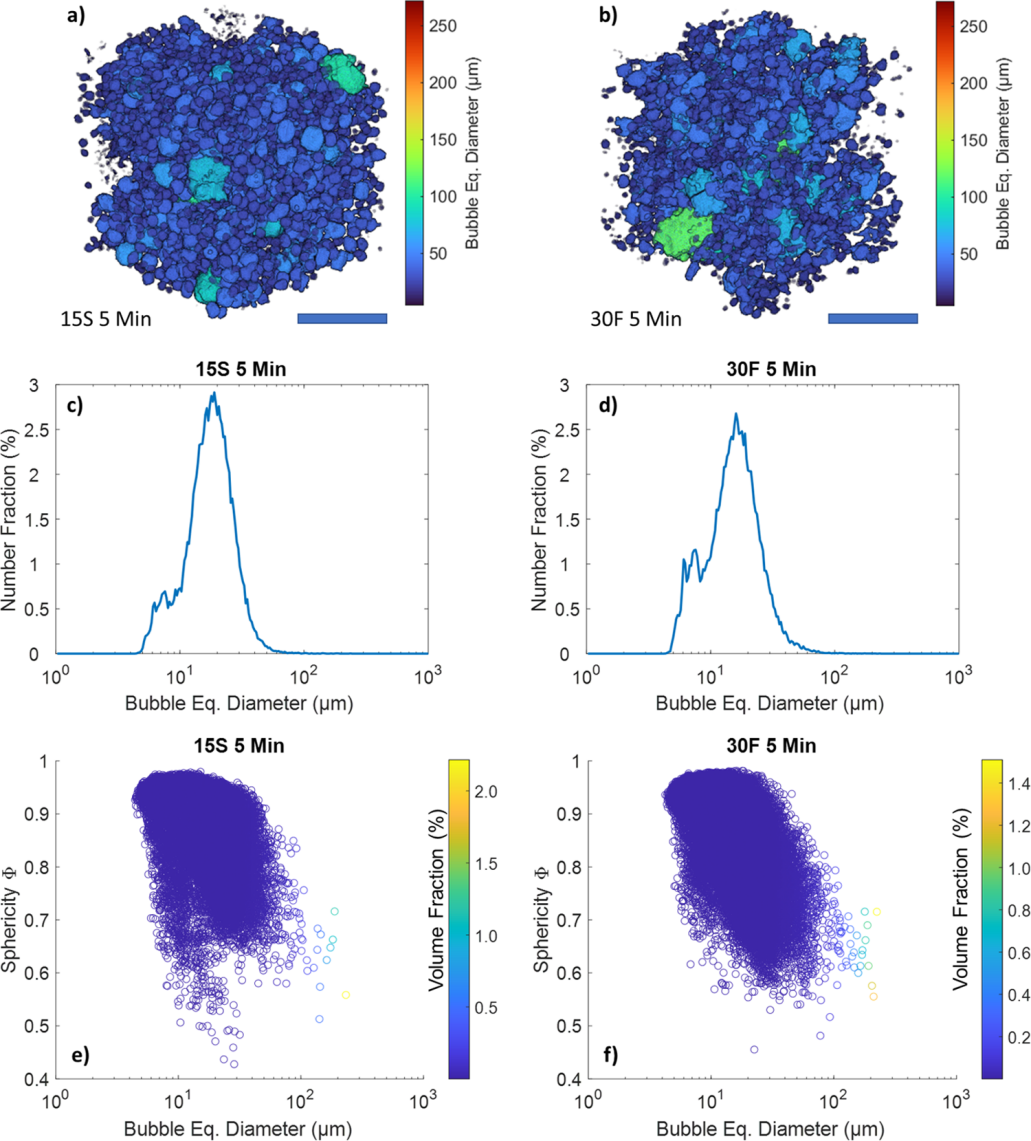
3D renderings of representative volumes of interest (VOIs) of sample 15S (a) and sample 30F (b) after 5 min of aeration. The scale bar is 250 μm. Bubble equivalent diameter distribution for samples 15S 5 min (c) and 30F 5 min (d). Corresponding scatter plots with bubble size and sphericity (e, f). The color bar shows the volume fraction occupied by each bubble.

The samples analyzed in this work exhibited a distribution of the oleogel thickness with two main peaks: a smaller one at *ca.* 10 ± 9 μm, which represents the size of the channels of oleogel surrounding the air bubbles, and a larger, broader peak centered at approximately 35 ± 25 μm, resulting from the presence of large domains of unwhipped oleogel in the samples (highlighted in Figure 1a by red arrows). By comparing samples 15S and 30F after 5 and 30 min of aeration (Figure 1a,c compared to Figure 1b,d), the peak centered at 35 μm decreased in intensity more significantly for sample 15S than for sample 30F. This observation agrees with Metilli et al., where 30% w/w CB oleofoams displayed a coarse microstructure even after vigorous whipping, with domains of oleogel still detectable both in optical microscopy images and with the naked eye.^17^ The dispersed gas phase is displayed as two representative VOIs and described in terms of bubble size and shape distribution in Figure 2.

The size distribution of the gas bubbles was bimodal for both samples, with a main peak centered at 20 ± 9 and 19 ± 11 μm for samples 15S and 30F, respectively (Figure 2c,d). A smaller shoulder in the distribution, centered at ca. 7 μm, was detected as well. By inspecting the scatter plots in Figure 2e,f, the bubble sphericity ranged between 0.60 and 0.95, with an average value of 0.88 ± 0.11 for both samples. These results are in line with previous observations from Binks and Marinopoulos, who reported an average bubble size between 20 and 30 μm for whipped pure cocoa butter, observed by optical microscopy.^16^

In contrast to other techniques, XCT allows the examination of the shape of 3D objects, particularly the sphericity. Air bubbles in foams tend to minimize their surface tension by assuming a spherical shape. However, the presence of Pickering and bulk crystals in the oleogel and at the interface of bubbles can affect their shape. For the oleofoams presented in this work, the non-spherical nature of the bubbles can be clearly seen from the volume renderings in Figure 2a,b and the tomography slices of Figure 1. Additionally, from the scatter plots of Figure 2e,f, it can be noted that large air bubbles (diameter ≥ 100 μm) displayed lower sphericity (Φ = 0.65) compared to the smaller ones. The non-spherical shape of bubbles is due to the presence of a jammed layer of adsorbed crystals at the air/oil interface.^16,17,22,30^ The lower sphericity of large bubbles compared to smaller one can be explained by considering the Laplace pressure inside a bubble, which decreases with increasing bubble diameter; therefore, larger bubbles tend to be more deformable than smaller ones.^9^ Hence, it is possible to use the sphericity value to qualitatively derive what forces are prevalent at the air/oil interface.^34,35^

The average bubble size of the cocoa butter-based oleofoams does not seem to be significantly affected by the amount of fat crystals (*i.e.*, solid fat content, SFC%), which was also reported by Gunes et al. and by Brun et al.^20,36^ This behavior could stem from the mechanical breakage of cocoa butter crystal aggregates into cocoa butter nanoplatelets (CNPs) of similar size during aeration. Therefore, the stabilizing crystals would have similar properties for samples with different SFC% values, leading to the same bubble size distribution. Nevertheless, the total amount of crystals is not modified by aeration; hence, samples with higher CB % w/w, such as sample 30F, will contain larger amounts of CNPs in the bulk, which can affect the rate of air incorporation and the thermodynamic stability of the oleofoam. Similarly, an effect of the SFC% on the sphericity distribution of the air bubbles was not observed.

For both samples (15S and 30F), the size distribution was not affected significantly by the aeration time; only a slight decrease in intensity of the peak at around 7 μm was observed for sample 30F (Figure S1 in the Supporting Information). No significant variations were observed in the sphericity distribution with increasing aeration time either (Figure S2 in the Supporting Information). This result implies that a characteristic bubble size (and shape) distribution is reached during the first 5 min of aeration, after which the main changes occurring in the oleofoam microstructure concerned the oleogel phase and the amount of incorporated air.

**Figure 3:**
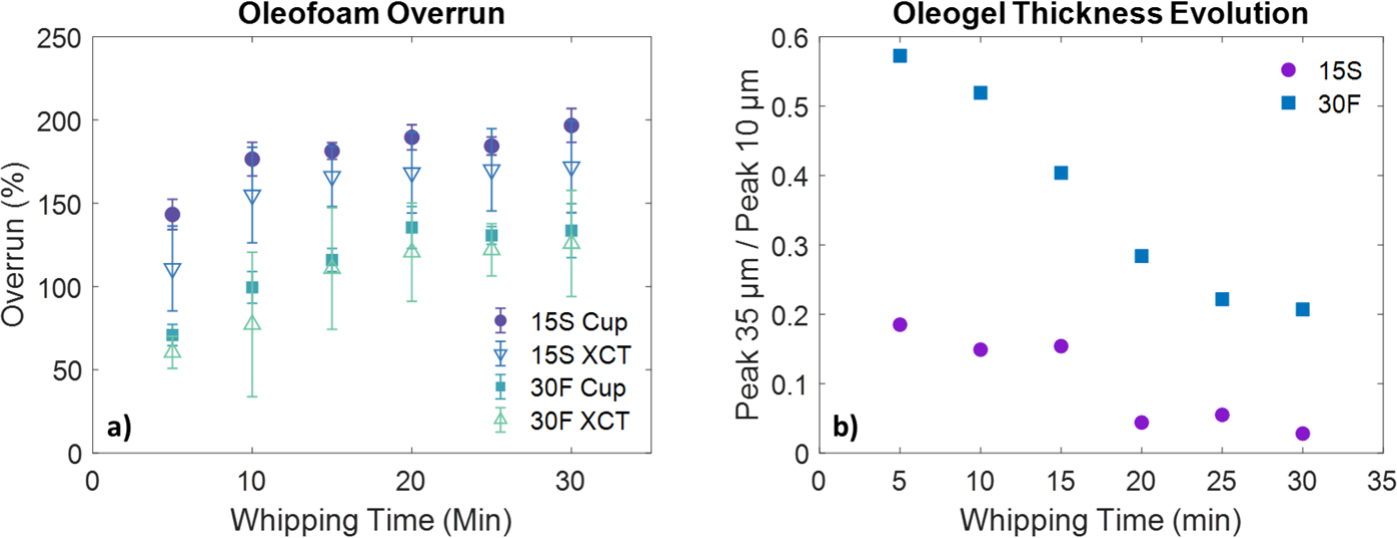
Evolution of the oleofoam overrun during whipping for samples 15S and 30F, as calculated from the cup method and XCT (a). Intensity ratio of the oleogel thickness peaks (35 μm vs 10 μm) during whipping (b).

The overrun calculated from XCT and by the cup method (described in the Experimental Section), and the evolution of the oleogel thickness during aeration for samples 15S and 30F are shown in Figure 3a,b, respectively.

Sample 15S displayed high overrun values (both via XCT and cup method) already after 5 min of aeration followed by a modest increase until reaching almost 200% overrun. Sample 30F, on the other hand, exhibited a lower initial overrun (70%), which increased more slowly than sample 15S until reaching a final value of 130%. The overrun values obtained from XCT were systematically lower than the overrun calculated by the cup method and displayed larger standard deviation values. It is hypothesized that the presence in the samples of air cavities larger than or of comparable size to the total volume analyzed with XCT (about 1 mm^3^) led to the underestimation of the overrun, as these could not be included in the tomography analysis. Additionally, the occurrence of air bubbles with comparable size to the VOI might have caused the large standard deviation in the XCT overrun (Metilli et al.^38^). Figure 3b displays the variation of oleogel thickness as the ratio between the intensity of the peak at 35 μm (i.e., the unwhipped oleogel domains) and the peak at 10 μm (i.e., the oleogel surrounding the air bubbles). Hence, the plot describes the depletion of the unwhipped oleogel domains during aeration. Waterfall plots showing the whole oleogel thickness evolution are also available in the Supporting Information (Figure S3). As already demonstrated in Figure 1, the amount of unwhipped oleogel was lower for sample 15S after 5 min, decreasing abruptly after 20 min of aeration. For sample 30F, on the other hand, the depletion of the oleogel phase followed a stepwise trend but starting from a higher amount of unwhipped oleogel, which persisted in the sample even after 30 min of aeration. In summary, 15S samples required less time to reach equilibrium in terms of overrun and microstructural homogeneity, whereas 30F samples required at least 30 min to reach a constant overrun. Nevertheless, these samples also contained unwhipped material that might affect their functionality and quality.

The results presented so far can provide an insight of the underlying mechanism of oleofoams aeration using a planetary mixer. Whipping breaks down fat crystals agglomerates to particles with similar properties (*i.e.*, size, shape, polymorphism), which act as Pickering stabilizers at the air/oil interface. Therefore, while maintaining a constant shear rate in the planetary mixer, the bubble size (and shape) distribution does not change significantly with the SFC%, or the prolonged aeration time, as reported also for aqueous foams.^38^ The effect of sample viscosity, which depends on the SFC%, is visible in the different air incorporation profiles: higher viscosity samples (30F) are characterized by a slower air incorporation and eventually a low final overrun, whereas low-viscosity samples (15S) present higher overruns, obtained during the first stages of aeration. Air incorporation is accompanied by the depletion of the oleogel domains, which are used as a source of fat crystals for stabilizing newly entrained air bubbles. This observation agrees with the findings of Mishima et al., where IR-probe microscopy was used to demonstrate that the amount of crystalline material in the bulk decreased with increasing aeration times.^15^

### Thermodynamic Stability of Oleofoams

**Figure 4:**
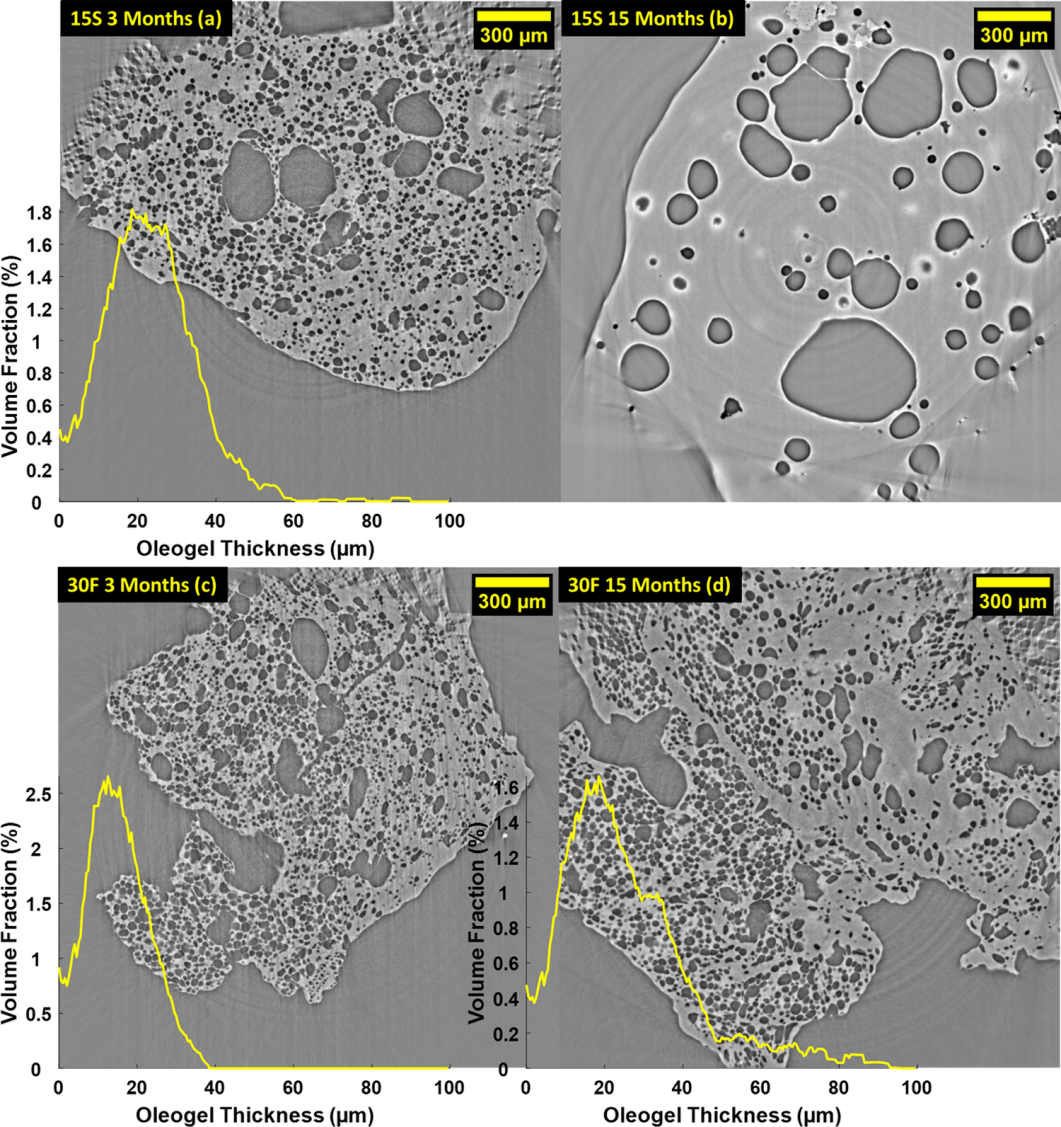
Comparison of the oleofoam microstructure during storage conditions for sample 15S 3 months (a), 15S 15 months (b), 30F 3 months (c), and 30F 15 months (d). The oleogel thickness distribution is overlaid on the respective tomography slices.

Figure 4 shows the microstructure of samples 15S and 30F after 3 and 15 months of storage at ambient temperature.

After 3 months (Figure 4a,c), both 15S and 30F oleofoams contained a lower amount of air bubbles and displayed a larger oleogel thickness, compared to their respective fresh samples in Figure 1. After 15 months of storage, however, sample 15S contained only few large bubbles (diameter 100–300 μm) (Figure 4b), whereas sample 30F (Figure 4d) retained a similar microstructure to after 3 months of storage. For this specific sample, however, the mean oleogel thickness increased further. From a macroscopic perspective, the aged oleofoam samples did not display significant oil drainage but their volume decreased, as shown in the Supporting Information (Figure S4). The samples exhibited large voids along the graduated cylinder, with visible fractures throughout the foam. Hence, it was not possible to determine accurately the decrease in overrun using volumetric measurements. Table 1 contains the main quantitative parameters describing the microstructure of the aged foams.

**Table 1:** Parameters Describing the Microstructure of Fresh and Aged Oleofoams (15S and 30F) in Comparison with Their Fresh Analogues

Sample	OR_XCT_	*D*_oleogel_	Bubbles/μm^3^ (× 10^–6^)	Mean *D*_eq_	Mean Φ
15S 30 min (fresh)	171.2 ± 26.0	11 ± 6	51.10 ± 5.97	20 ± 9	0.86 ± 0.11
15S 3 months	50.0 ± 7.5	25 ± 10	20.16 ± 2.20	21 ± 10	0.93 ± 0.08
15S 15 months	13.6 ± 9.4	n/a	1.28 ± 0.65	27 ± 19	0.86 ± 0.15
30F 30 min (fresh)	125.8 ± 31.2	20 ± 13	46.45 ± 5.52	19 ± 11	0.88 ± 0.11
30F 3 months	71.5 ± 12.5	18 ± 8	30.90 ± 1.87	18 ± 8	0.92 ± 0.09
30F 15 months	69.7 ± 35.7	26 ± 12	22.85 ± 7.47	24 ± 10	0.92 ± 0.07

In agreement with what observed in Figure 4, the increase in the mean oleogel thickness was larger for sample 15S than sample 30F, after 3 months. For sample 15S at 15 months, it was not possible to determine the oleogel thickness from image analysis as this was comparable in length with the edge of the sampling VOI (500 μm). After 15 months of storage, sample 30F displayed a further increase in the thickness of the oleogel phase. At the same time, the decrease in overrun calculated from XCT was more significant for sample 15S (from 170 to 50%) compared to sample 30F (from 125 to 75%). After 15 months, sample 15S barely contained any air (10% overrun), whereas sample 30F maintained a similar overrun to the 3 months sample, albeit with larger standard deviation. The normalized number of bubbles for each VOI decreased by 60% for sample 15S and by 34% for sample 30F after 3 months. After 15 months, very few bubbles are still present in the 15S samples, while the normalized number of bubbles per VOI for the 30F shows only a moderate decrease.

**Figure 5:**
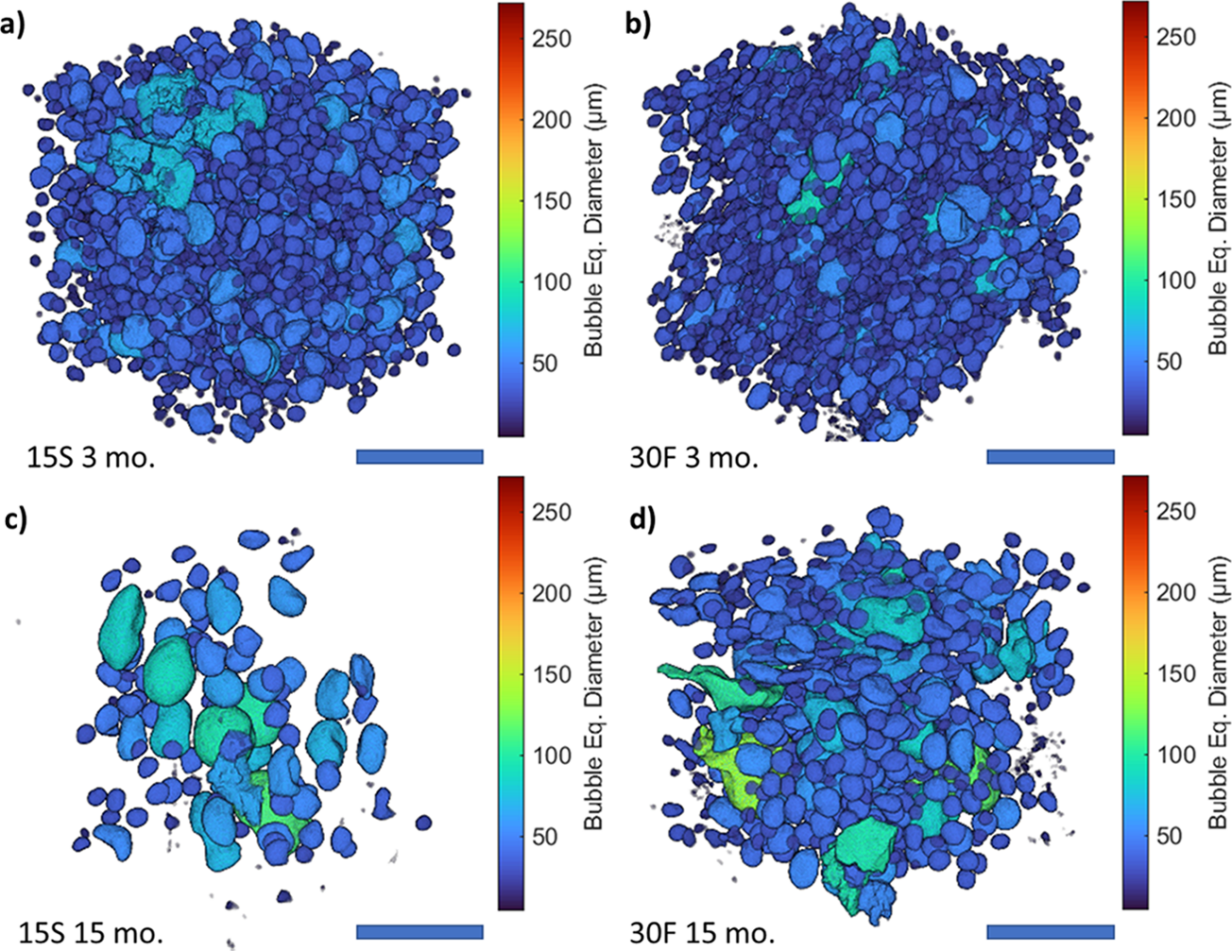
3D renderings of representative VOI for each of the aged oleofoam samples: 15S 3 months (a), 30F 3 months (b), 15S 15 months (c), and 30F 15 months (d). The scale bar is 250 μm.

**Figure 6:**
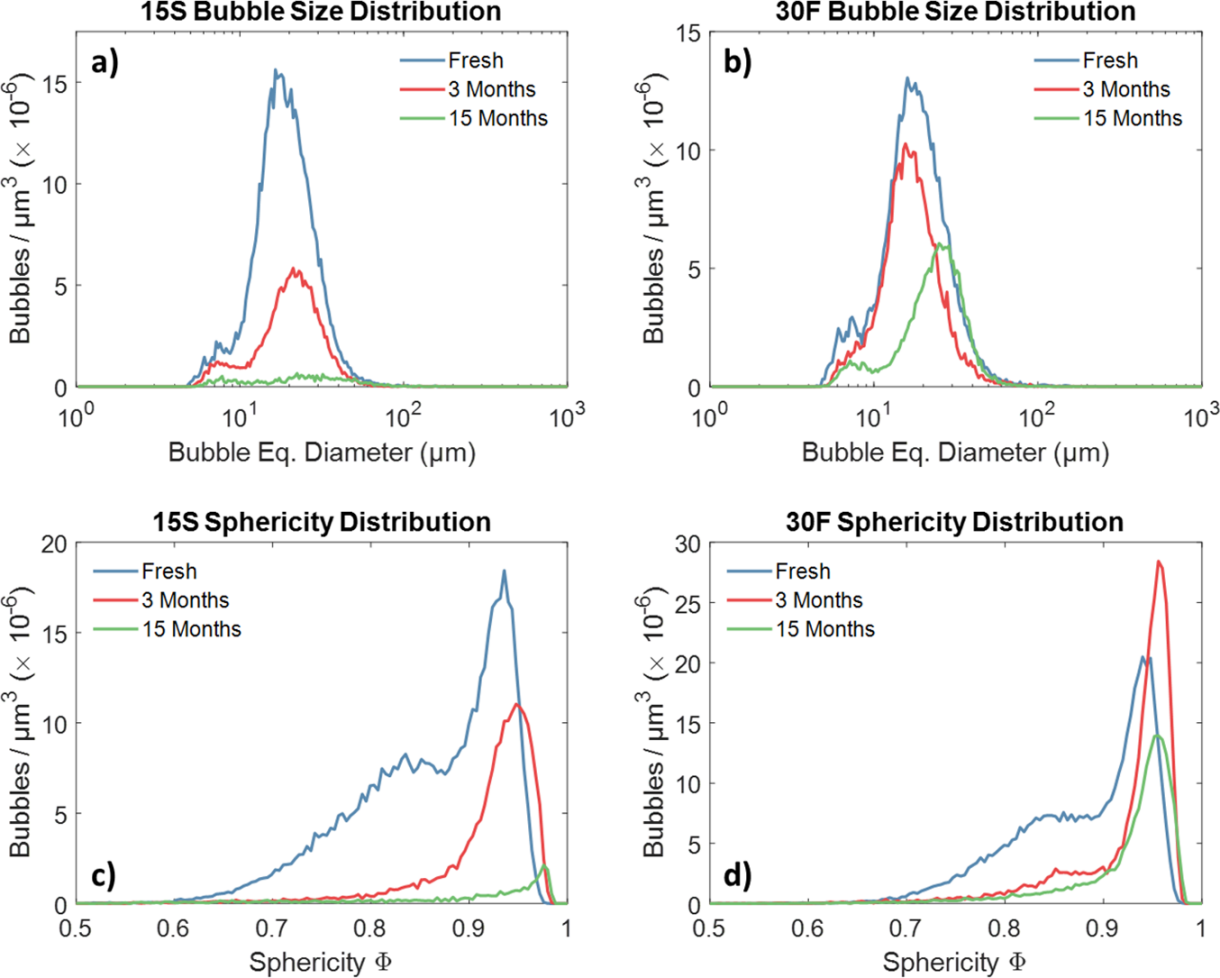
Volume-normalized bubble size distribution for 15S (a) and 30F (b) fresh samples and after 3 and 15 months of storage at 20 °C. Bubble sphericity distribution of the samples is shown for sample 15S (c) and 30F (d).

Therefore, higher SFC% seems to be beneficial to oleofoam stability.^20,22^ 3D renderings of the dispersed gas phase for the aged samples are shown in Figure 5; the bubble size and shape distributions are displayed in Figure 6, in comparison with the fresh samples.

The evolution of the size distribution over time has a more interesting trend compared to the numerical values shows in Table 1. In fact, the size distributions of Figure 6 clearly show the persistence of several small sized bubbles (peak centered at ca. 7 μm) during storage up to 15 months, even though in a decreasing number. This is more evident for the 30F sample than the 15S. The size of most of the bubbles in both samples does not change significantly in the first 3 months; however, a moderate increase in size is observed between 3 and 15 months. The average sphericity of the bubbles increased to 0.93 ± 0.08 and 0.92 ± 0.09 for samples 15S and 30F, respectively, after 3 months. These values remain fairly constant after 15 months.

All the discussed quantitative variations in the air phase properties are visible by qualitatively comparing the volume renderings of Figure 2 with the ones shown in Figure 5. From these results, it is evident that oleofoams are subjected to post-processing physical phenomena that affect significantly their microstructure and therefore the overall properties of the material.

The decrease in the measured overrun and in the normalized number of bubbles per VOI implies that bubbles have left the oleofoam samples by either diffusion to the samples’ surface, or to air bubbles whose volume exceeds the size of the VOI or the field of view of the XCT setup. The latter phenomenon is due to Ostwald ripening and coalescence of air bubbles. The disappearance of smaller bubbles combined with the inability to detect very large air domains with the XCT protocol explains why the average size of the majority of the air bubbles does not change significantly during storage. To achieve a better estimation of the microstructure of aged oleofoams, the presented measurements could be integrated with XCT that uses a larger field of view, where larger air bubbles or voids are detectable. The persistence of small air bubbles during storage could be ascribed to effect of Pickering stabilization. In fact, adsorbed fat crystals at air/oil interfaces can prevent air diffusion and hence disproportionation of air bubbles.^1,2^

The changes in the sphericity of the air bubbles are likely related to Ostwald ripening of the fat crystals, with small CNPs dissolving or incorporating in large crystalline aggregates. This was observed with polarized microscopy of both aged samples, which displayed larger quantities of birefringent fat crystals in the bulk after 3 and 15 months (Figure S5 in the Supporting Information) compared to the fresh samples. Ostwald ripening of crystals is possibly facilitated by the decrease in overrun of the oleofoams, which increases oleogel thickness. The observed increase in the average sphericity of air bubbles during storage could result from the dissolution of the smaller CNPs attached at the air/oil interface, which caused the air bubble boundary to relax. In addition to the effect of CNP crystals, the decrease in the bubble number density might also contribute to the relaxation of the surface of the surviving bubbles.

Analysis of the viscoelastic profile of the aged samples showed that storage resulted also in a slight increase in the loss modulus (G″) of oleofoams, as shown in Figure S6 in the Supporting Information. After 15 months of storage, only samples with higher amounts of stabilizing crystals (i.e., higher SFC%) are able to retain an aerated structure. Therefore, the amount of fat crystals plays a significant role in the stability of the oleofoams. At this stage of aging, however, the effect of disproportion was indeed obvious, with a visible increase in the average bubble size.

### Evolution of Oleofoams’ Microstructure during Heating

**Figure 7:**
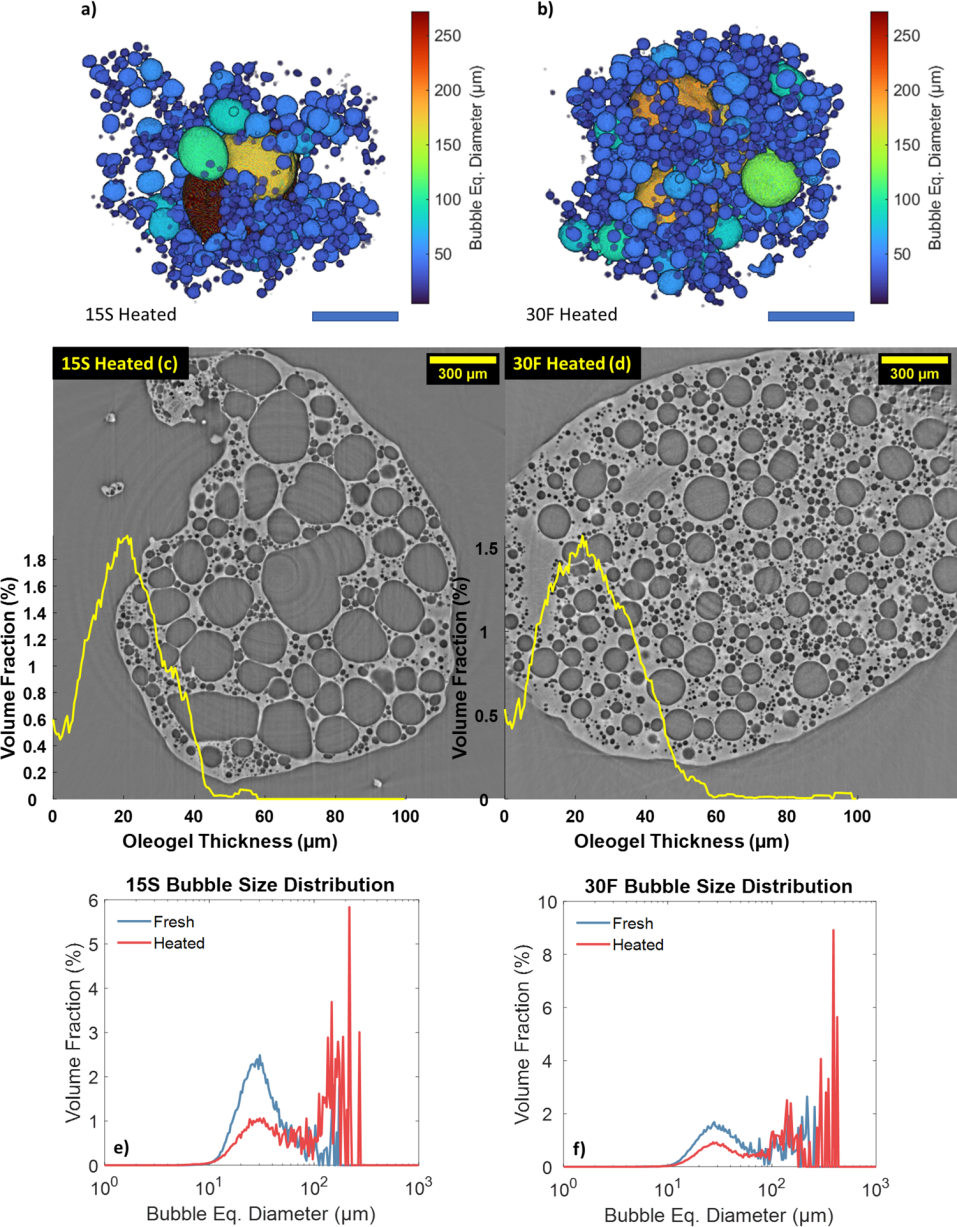
3D renderings (a, b) and tomography slices (c, d) of samples 15S and 30F after being heated. The oleogel thickness distribution is overlaid on the tomography images. The scale bar for the 3D renderings is 250 μm. Volume-weighed size distributions of sample 15S heated (e) and 30F heated (f) compared with their respective fresh samples.

Figure 7 shows the microstructure of fresh samples (15S and 30F) after being heated to 25 and 27 °C, respectively.

Heating oleofoams caused partial melting of the fat crystals surrounding the air bubbles, as well as the ones in the bulk, resulting in the coalescence of the air bubbles and the relaxation of the surface of the air bubbles to a more spherical shape. The oleogel thickness of both samples increased compared to the respective fresh samples; however, in contrast with aged samples, such an increase was due to the dissolution of the air bubbles, rather than the growth of the crystals in the continuous phase. By comparing Figure 7a,c with Figure 7b,d, sample 15S displayed larger coalesced bubbles, potentially due to the lower amount of fat crystals in the bulk, which can prevent aggregation of neighboring bubbles. Table 2 summarizes the changes in the microstructure of heated samples.

**Table 2:** Parameters Describing the Microstructure of the Heated Samples (15S and 30F) Compared to Their Fresh Analogues

Sample	*D*_oleogel_	Bubbles/μm^3^ (× 10^–6^)	Mean *D*_eq_	*D*[4,3]	Mean Φ
15S fresh	11 ± 6	51.1 ± 5.97	20 ± 9	45 ± 37	0.86 ± 0.11
15S heated	20 ± 26	20.49 ± 5.41	18 ± 12	105 ± 74	0.93 ± 0.06
30F fresh	20 ± 13	46.45 ± 5.52	19 ± 11	78 ± 69	0.88 ± 0.11
30F heated	23 ± 33	23.58 ± 15.15	19 ± 13	164 ± 136	0.95 ± 0.05

During the thermal treatment, the samples became less viscous, flowing partially out of the field of view of the X-ray beam. Hence, a direct comparison of the fresh and heated samples could not be made. In fact, the calculated overrun for the heated samples resulted higher (> 200%) compared to the fresh samples. As no air was further incorporated during heating, this observation could be explained only with a partial loss of the continuous phase. Nevertheless, a qualitative description of the effect of heating on the oleofoam microstructure could be carried out. The normalized amount of air bubbles decreased more significantly for sample 15S heated, with a similar trend observed during aging. The sphericity of the air bubbles increased for both samples, as visible in Figure 7a–d.

Analysis of the volume-weighed size distribution was more helpful in describing the changes in microstructure for the heated samples. In particular, the main distribution peak, centered at 30 μm, decreased in favor of several peaks between 100 and 500 μm, which represented the coalesced air bubbles. In fact, the volume-weighed mean diameter *D*[4,3], increased by 130% for sample 15S, and by 109% for sample 30F. The effect of heating is also visible in the scatter plots of Figure S7 in the Supporting Information, with a shift in the bubble population toward higher sphericity values, compared to the fresh samples from Figure 2. In particular, coalesced bubbles with diameters exceeding 100 μm and high sphericity are visible in the top-right area of the plot. The melting of the stabilizing CNPs, both at the interface and in the continuous phase, caused the oleofoam to behave more like a liquid foam.

**Figure 8:**
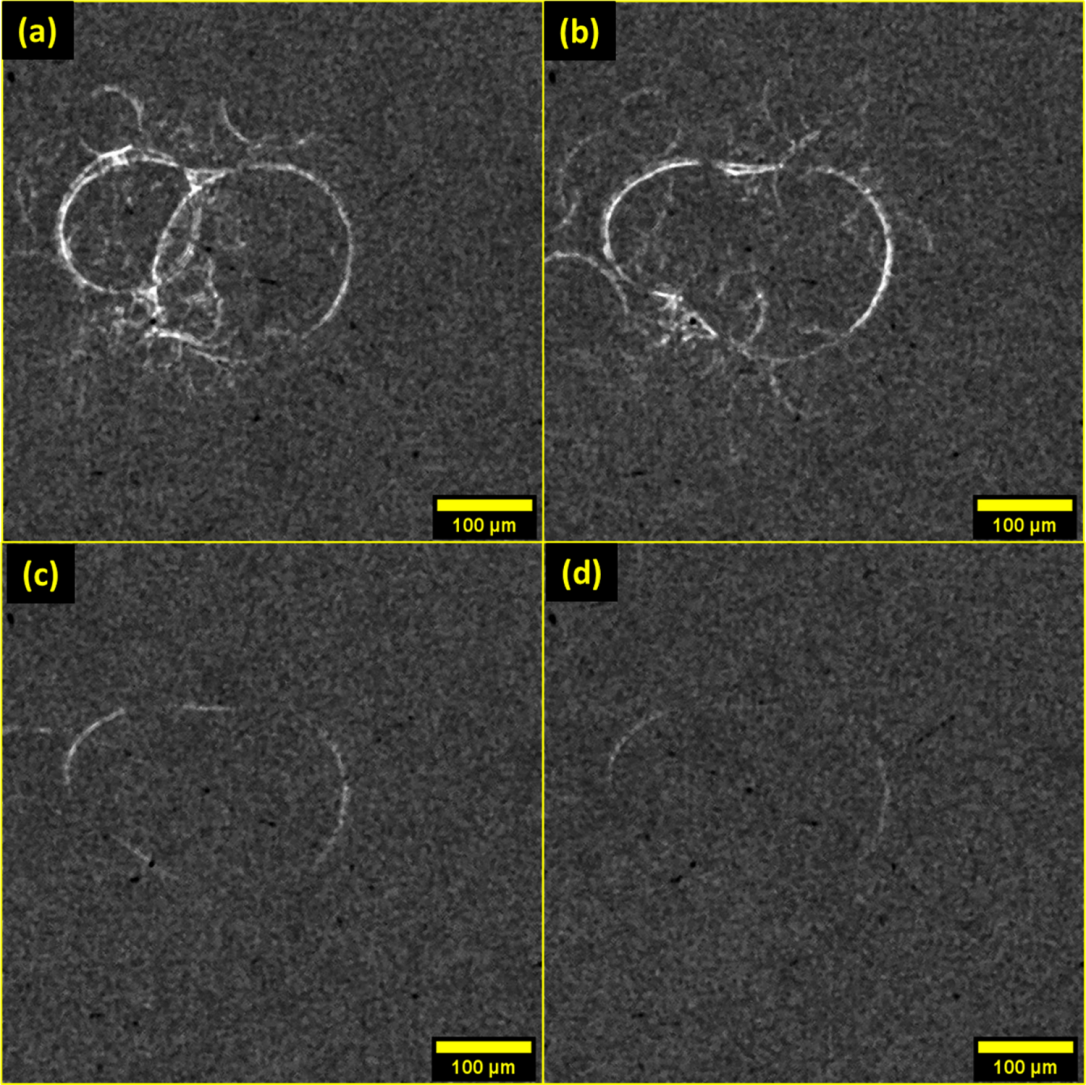
Sequence of difference images, obtained from XRR, showing the coalescence of two neighboring bubbles from a 30F fresh sample during thermal treatment.

In order to study the destabilization mechanism that caused the changes in the microstructure of the heated samples, the sequence of XRR frames was analyzed. Figure 8 contains selected difference image frames of a 30F sample during heating.

During heating, the main events captured involved either macroscopic sample movement, as the partially melted oleogel phase became less viscous, or bubble coalescence, which occurred by sequential merging of neighboring bubbles. More specifically, bubbles were seen to aggregate (Figure 8a), with consequent film rupturing and merging (Figure 8b) followed by bubble shape relaxation (Figure 8c,d).^39^ From this analysis, it can also be appreciated that bubble aggregation was irreversible, and that film rupturing and merging occurred on a shorter timescale (ca. 0.5 s) compared to the relaxation of the newly coalesced air bubble. It is highly likely that coalescence was accompanied by Ostwald ripening of the air bubbles, considering that during heating, the CNPs that act as Pickering stabilizers melt, leaving no physical barrier to prevent gas diffusion between bubbles. However, the shrinkage of air bubbles could not be observed unambiguously from the XRR difference images. This could be ascribed to the high level of noise in the difference image stack, as bubble shrinkage affects mainly small bubbles rather than large, more visible ones.

**Figure 9:**
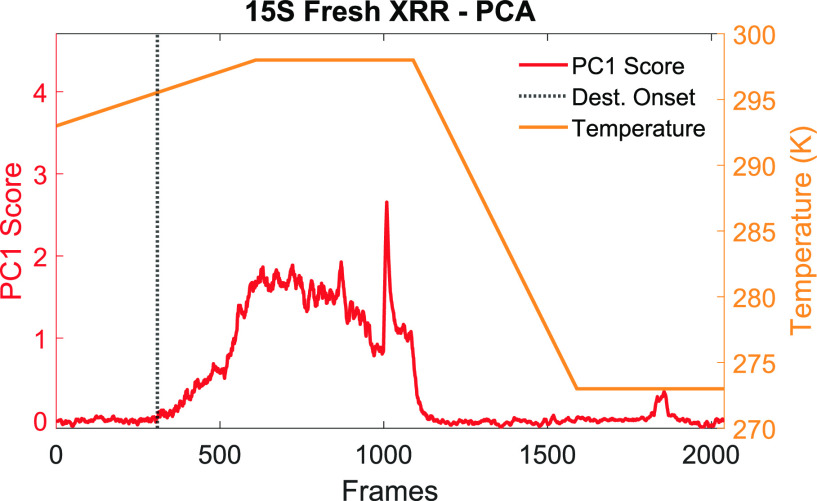
PCA score plot of the difference image stack collected during heating of sample 15S (red), together with the temperature profile during the thermal treatment (orange) The onset of destabilization (*T*_onset_) is shown with the black dotted line.

The occurrence of coalescence events in the difference image stack can be monitored to determine the corresponding temperature at which the oleofoam begins to destabilize. To do so, the sequence of XRR images was analyzed using principal component analysis. The score of the first principal component (PC1), which accounts for most of the variance in the image sequence dataset, was plotted against the temperature to establish precisely the onset of microstructural destabilization in the oleofoam samples. The results are shown in Figure 9 for a 15S sample.

Changes in the pixel intensity caused by either sample movement or bubble coalescence are shown as an abrupt increase of the PC1 score value (red trace). In particular, a significant deviation of the PC1 score from the baseline during heating was attributed to the onset of mechanical destabilization of the oleofoam sample. The corresponding temperature onset (XRR *T*_onset_) is shown in Figure 9 as a dotted black line. Comparison of the data provided from XRR with DSC of the same samples demonstrated that the oleofoam microstructure is subject to changes after the melting onset (DSC *T*_onset_) and that the destabilization is promptly halted as soon as the temperature is lowered below the DSC *T*_melting_. Table S1 in the Supporting Information lists the melting parameters, calculated from DSC, and the XRR *T*_onset_ for samples 15S and 30F.

This type of analysis suggests that structural destabilization in oleofoams occurred at higher temperatures compared to the onset of crystal melting. In particular, the difference in onset temperature (Δ*T*_onset_) demonstrated that sample 30F exhibited a larger thermal delay in its mechanical destabilization than sample 15S, confirming the higher stability endowed by the higher concentration of fat crystals. Furthermore, the air phase could also act as a thermal insulator, slowing the heat transfer toward the center of the sample and causing a delay in the melting of the crystals.

## Conclusions

In this paper, the three-dimensional native microstructure of fat-stabilized oleofoams was investigated for the first time, both in static and dynamic experiments, using X-ray tomography and radiography. The aeration of oleofoams proceeded with a gradual increase in volume, concomitant with a decrease in the continuous phase thickness. Low-cocoa butter samples displayed a higher overrun (170%) and a lower oleogel thickness (10 μm), reaching an overrun equilibrium value after 15 min of whipping. High-cocoa butter samples, on the other hand, incorporated less air (125% overrun) and featured a coarse final microstructure, retaining fragments of unwhipped oleogel. The air bubble size distribution, centered at 20 μm, was not affected by the amount of solid fat or by the whipping time, suggesting that the shear induced during aeration produced stabilizing crystals with similar properties, regardless of the sample composition and the duration of whipping. Both samples contained a small number of large, deformed bubbles, potentially resulting from coalescence during air entrainment.

Oleofoam samples showed significant structural changes during storage, with the oleogel phase increasing its thickness, while the overrun decreased significantly for both samples analyzed. The mean sphericity of the air bubbles increased due to Ostwald ripening of the fat crystals. The higher concentration of solid fat in the sample contributed to slowing down the disproportionation of the air bubbles, with sample 30F retaining a similar size distribution profile after 3 months. After prolonged storage conditions, however, only samples with 30% w/w CB maintained an aerated structure. Therefore, higher amounts of stabilizing crystals were beneficial to retaining the overrun and counteracting phase separation. Ostwald ripening of the gas bubbles, together with the persistence of strongly Pickering stabilized small bubbles, was also observed for these samples.

Heating the oleofoam samples to their melting point resulted in an increase in bubble sphericity, bubble coalescence, oleogel thickness, and a reduction of the total number of air bubbles. Sample 15S was more prone to coalescence than sample 30F, potentially due to the lower amount of stabilizing crystals. The increase in the bubble size followed by melting of the crystals both at the interface and in the bulk support the hypothesis that bulk contribution to stability in oleofoams is fundamental to prevent gas diffusion. The dynamic changes in the oleofoam microstructure were captured for the first time with XRR, showing clear evidence of bubble coalescence during heating. XRR data combined with statistical analysis also provided a mechanical destabilization parameter, XRR *T*_onset_, which gives a more accurate temperature for the mechanical collapse of the oleofoams as compared with traditional DSC data.

This body of work, owing to the non-invasive, three-dimensional approach to the study of oleofoams, contains significant information on the physical behavior of these emerging materials, in relation to relevant processes such as their aeration, storage conditions that will contribute to their understanding, and use in material formulation.

## A. APPENDIX

Table showing the descriptors used to describe the microstructure of oleofams (Table A1).

**Table A1:** Descriptors Used to Describe the Microstructure of Oleofoams Obtained from each VOI

descriptor	abbreviation	equation
bubble equivalent diameter	*D*_eq_	
bubble sphericity	Φ	
volume-weighted mean bubble diameter	*D*[4,3]	
overrun calculated from XCT	OR_XCT_	
oleogel thickness	*D*_oleogel_	calculated with BoneJ using Hildebrand & Rüegsegger (1997) method

## Supporting Information Available

Bubble size distribution of oleofoams at different aeration times (Figure S1), bubble sphericity distributions of oleofoams at different aeration times (Figure S2), oleogel thickness distribution of oleofoams at different aeration times (Figure S3), microstructure of oleofoams, observed in graduated cylinders at different storage times (Figure S4), thickness of the continuous oleogel phase, observed by PLM at different storage times (Figure S5), results for oscillatory rheology experiments of aged oleofoam samples (Figure S6), bubble size and shape distributions of oleofoams before and after heating (Figure S7), comparison of the melting properties of oleofoam samples calculated from XRR and DSC (Table S1) (PDF)

## ABBREVIATIONS

VOI: volume of interest
OR: overrun
XCT: X-ray tomography
XRR: X-ray radiography
