# Supplementary material for: Application of X-ray Microcomputed Tomography for the Static and Dynamic Characterization of the Microstructure of Oleofoams
Source: Langmuir. 2022 Jan 20;38(4):1638–50. doi: 10.1021/acs.langmuir.1c03318 (PMC8812118; doi:10.1021/acs.langmuir.1c03318)
Supplement: Supplementary file 1 — la1c03318_si_001.pdf [file la1c03318_si_001.pdf]

Supporting Information

Application of X-ray microcomputed tomography for the static and dynamic characterization of the microstructure of oleofoams

Lorenzo Metilli[1], Malte Storm[2,3], Shashidhara Marathe[2], Aris Lazidis[4], Stephanie Marty-Terrade[5] and Elena Simone[1,6]*

[1]School of Food Science and Nutrition, Food Colloids and Bioprocessing group
University of Leeds
Woodhouse Lane, Leeds LS29JT, UK

[2]Diamond Light Source Ltd.
Harwell Science and Innovation Campus, Didcot, OX110DE, UK

[3]Helmholtz-Zentrum hereon
Max-Planck-Str 1, 21502 Geesthacht, Germany

[4]Nestlé Product Technology Centre Confectionery
Haxby Road, York YO31 8TA, UK

[5]Nestlé Research
Vers-chez-les-Blanc, 1000 Lausanne 26, Switzerland

[6]Department of Applied Science and Technology (DISAT)
Politecnico di Torino, Corso Duca degli Abruzzi 24, Torino, Italy

1. Effect of aeration time on the bubble size distribution of oleofoams

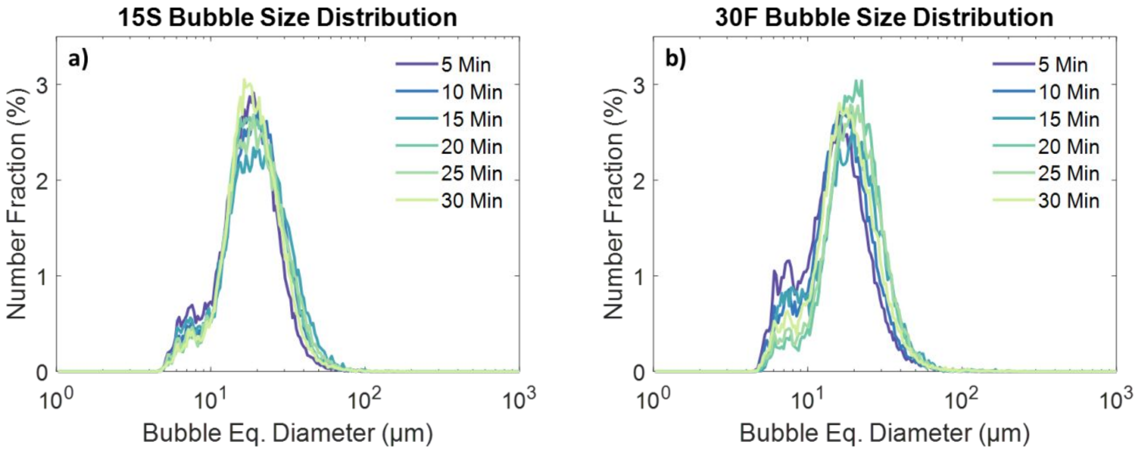

Figure **S1**. Evolution of the bubbles' equivalent diameter number distribution during aeration for sample 15S (a) and sample 30F (b).

2. Effect of aeration time on the bubble sphericity distribution of oleofoams

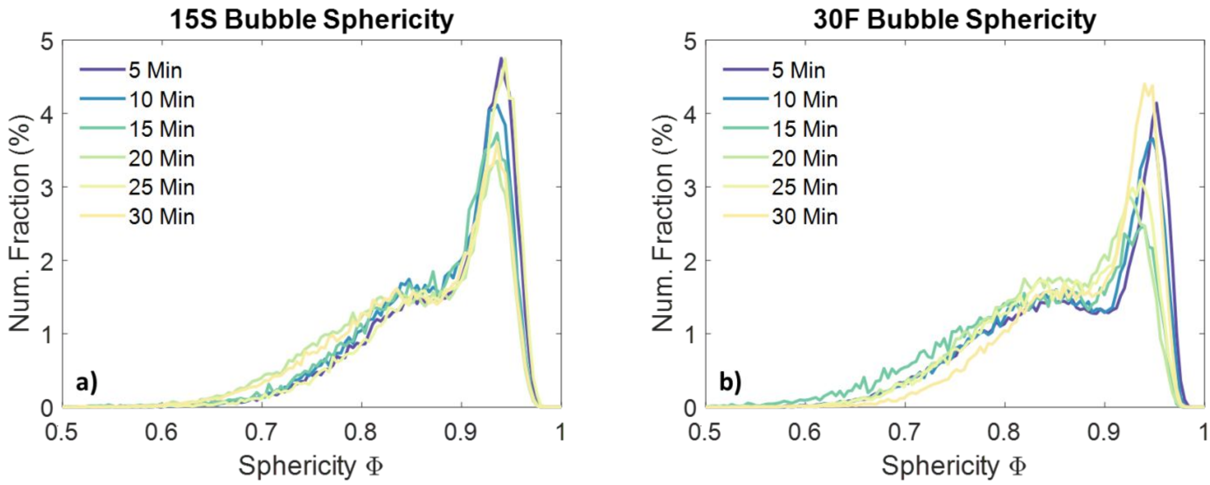

Figure S2. Evolution of the bubble sphericity during aeration for sample 15S (a) and 30F (b).

3. Effect of aeration time on the oleogel thickness distribution of oleofoams

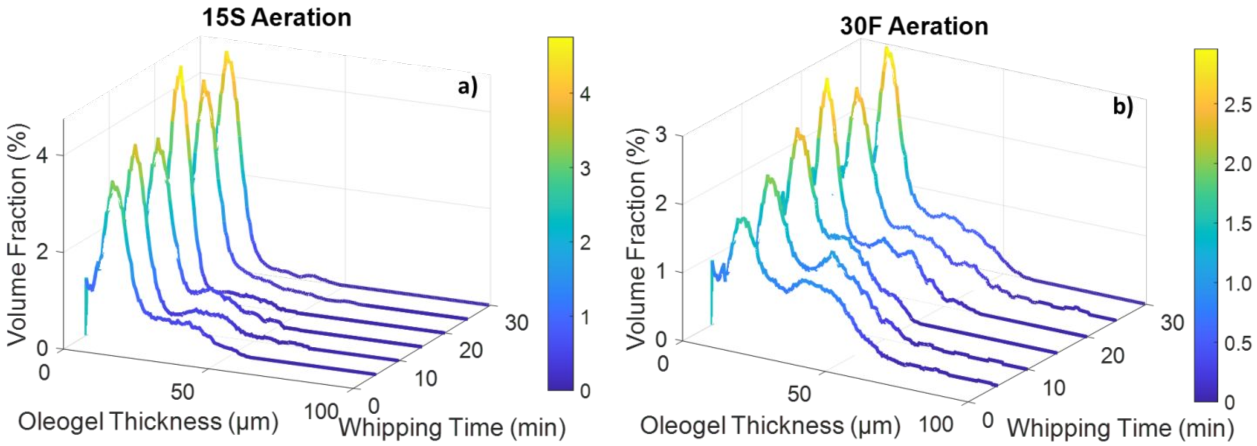

Figure S3. Waterfall plots displaying the evolution during aeration of the oleogel phase distribution for sample 15S (a) and sample 30F (b).

4. Effect of storage time on the microstructure of oleofoams, observed in graduated cylinders

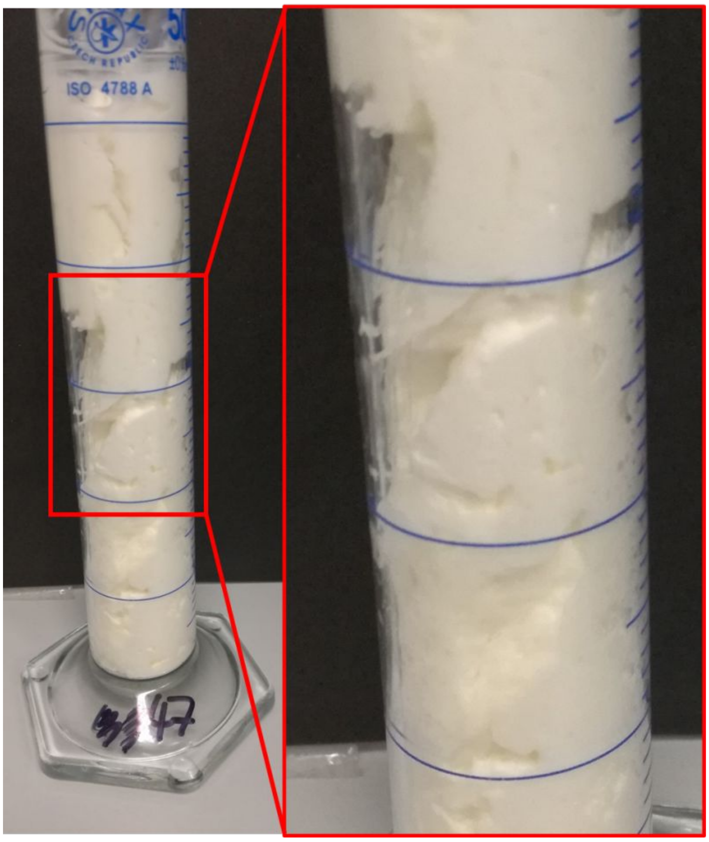

Figure S4. Sample 15S after three months of storage at 20°C. The magnification shows the internal fracture in the sample, with consequent loss of the air phase.

5. Effect of storage time on the thickness of the continuous oleogel phase, observed by PLM.

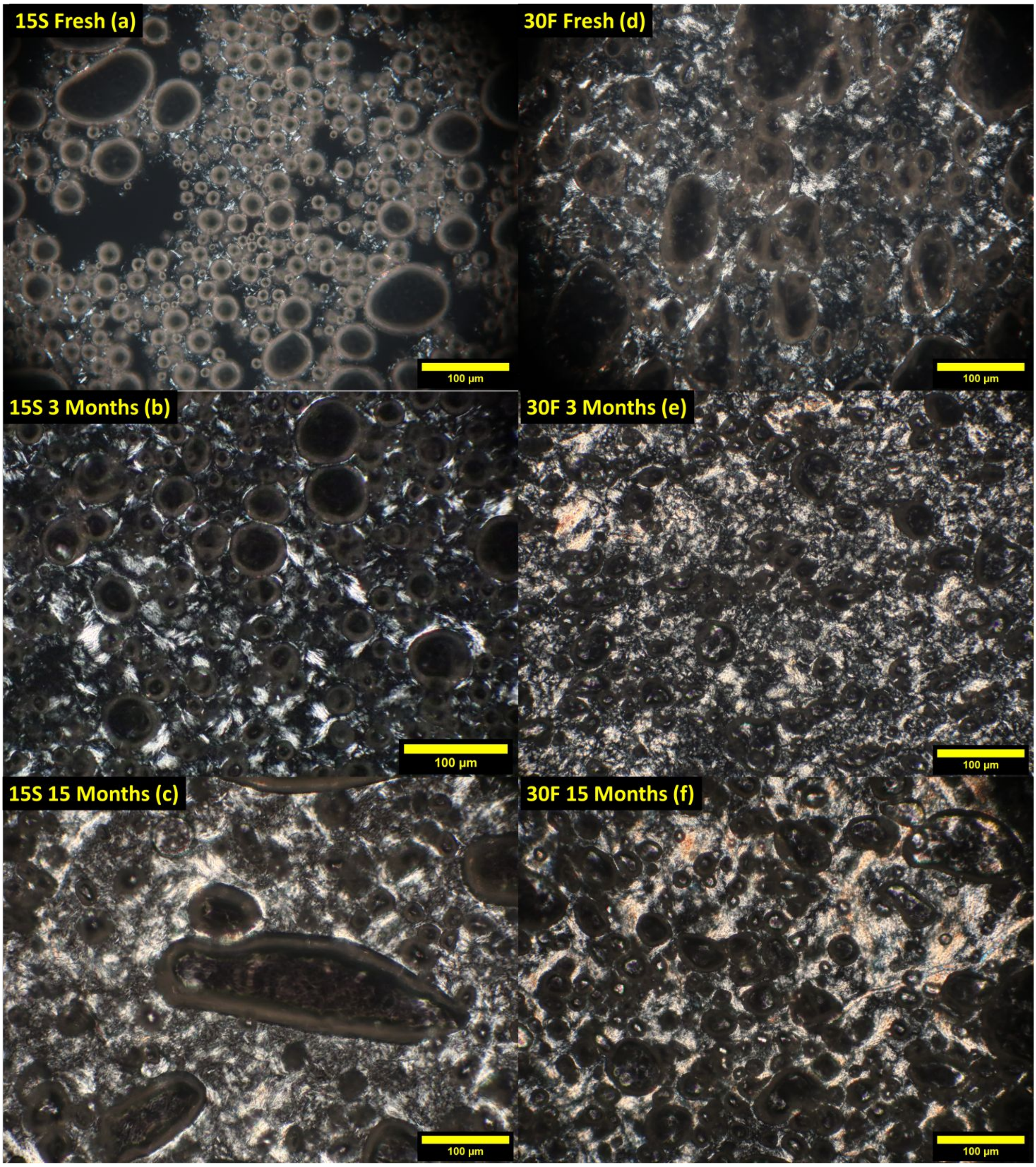

Figure S5. Polarized light images of oleofoam samples at different storage times: 15S fresh (a), after 3 months (b) and after 15 months (c); sample 30F fresh (d), after 3 months (e) and after 15 months (f).

6. Oscillatory rheology experiments of aged oleofoam samples.

Aged oleofoam samples were investigated with oscillatory rheology with an amplitude sweep experiments. The measurements were carried out on a MCR 302 stress-controlled rheometer (Anton Paar, Austria) using a 25 mm parallel plate, and a sample gap of 1.0 mm. The amplitude sweeps were applied between 0.001% and 10% strain, with a fixed frequency of 1 Hz. The temperature was set to 20°C and maintained using a Peltier hood connected to a F25-HE water circulator (Julabo, Germany). The samples were investigated after 15 months of storage at 20°C, in triplicates. Data analysis was carried out in the Rheocompass version 1.21 software (Anton Paar, Austria), and compared with the results from fresh oleofoam samples.

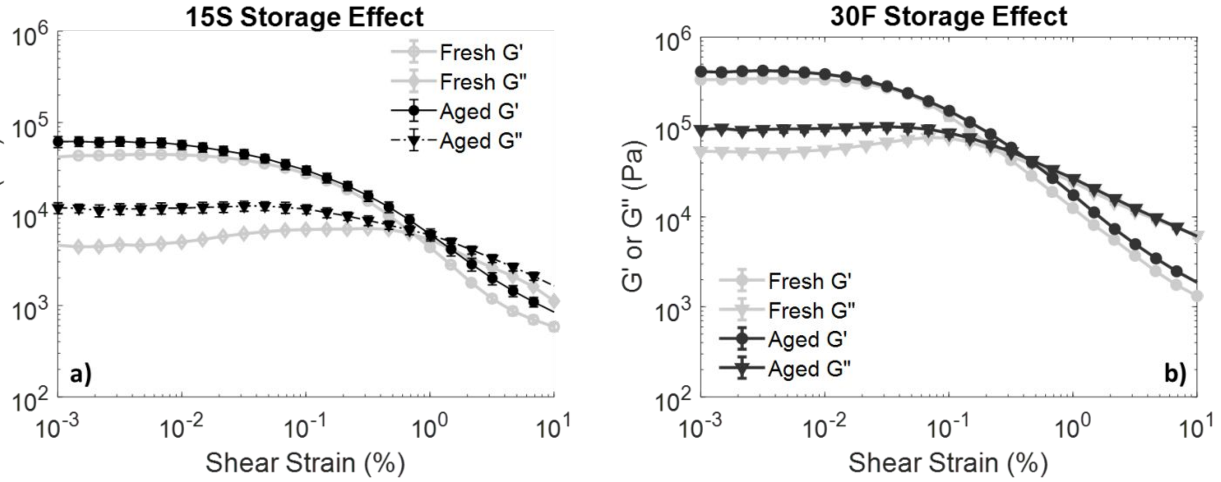

Figure S6. Elastic modulus (G') and viscous modulus (G") of 15S samples fresh and aged 15 months (left), 30F samples fresh and aged 15 months (right).

7. Effect of heating on the bubble size and shape distribution.

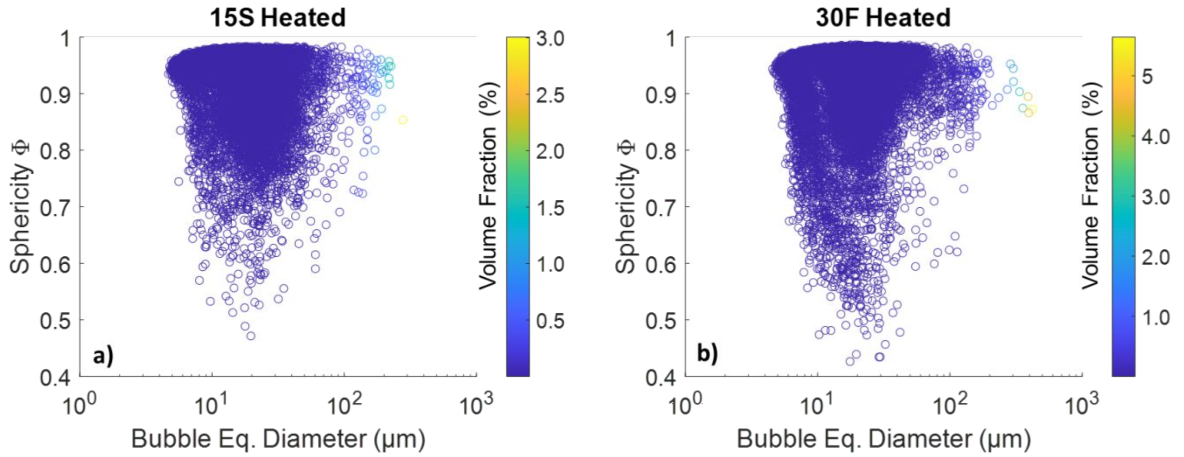

Figure S7. Scatter plot showing the size and sphericity distribution of the air phase for sample 15S Heated (a) and 30F Heated (b).

8. Comparison of the melting properties of oleofoam samples calculated from XRR and DSC.

Table S1. Onset temperatures of melting for fresh oleofoam samples as calculated from XRR and DSC.

Sample	DSC T_{onset} (˚C)	DSC $T_{melting}$ (˚C)	XRR T_{onset} (˚C)	ΔT_{onset}
15S Fresh	21.28 ± 0.27	24.98 ± 0.67	23.53 ± 0.50	2.25
30F Fresh	21.87 ± 0.82	27.30 ± 0.08	25.21 ± 0.58	3.3
